# Rare α^0^-thalassemia deletions detected by MLPA in five unrelated Brazilian patients

**DOI:** 10.1590/1678-4685-GMB-2016-0330

**Published:** 2017-10-02

**Authors:** Natália O. Mota, Elza M. Kimura, Roberta D. Ferreira, Gisele A. Pedroso, Dulcinéia M. Albuquerque, Daniela M. Ribeiro, Magnun N. N. Santos, Cristina M. Bittar, Fernando F. Costa, Maria de Fatima Sonati

**Affiliations:** 1 Laboratório de Hemoglobinopatias, Departamento de Patologia Clínica, Faculdade de Ciências Médicas, Universidade Estadual de Campinas (UNICAMP), Campinas, SP, Brazil.; 2 Hemocentro, Universidade Estadual de Campinas (UNICAMP), Campinas, SP, Brazil.; 3 hospital de Clínicas de Porto Alegre, Porto Alegre, RS, Brazil.

**Keywords:** α-Thalassemia, Hb H disease, multiplex ligation-dependent probe amplification, MLPA, Brazilian population

## Abstract

Alpha-thalassemias are among the most common genetic diseases in the world. They are characterized by hypochromic and microcytic anemia and great clinical variability, ranging from a practically asymptomatic phenotype to severe anemia, which can lead to intrauterine or early neonatal death. Deletions affecting the α-globin genes, located on chromosome 16p13.3, are the main causes of α-thalassemia. Multiplex ligation-dependent probe amplification (MLPA) can be used to detect rearrangements that cause α-thalassemia, particularly large deletions involving the whole α cluster and/or deletions in the HS-40 region. Here, MLPA was used to investigate the molecular basis of α-thalassemia in five unrelated patients, three of whom had Hb H disease. In addition to the -α^3.7^ deletion identified in the patients with Hb H disease, four different α^0^ deletions removing 15 to 225 kb DNA segments were found: two of them remove both the α genes, one affects only the regulatory element (HS-40) region, and another one extends over the entire α cluster and the HS-40 region. These results illustrate the diversity of α-thalassemia deletions in the Brazilian population and highlight the importance of molecular investigation in cases that present with microcytosis and hypochromia without iron deficiency and normal or reduced Hb A_2_ levels_._.

Thalassemias are among the most frequently found genetic diseases in populations. They are caused by mutations that affect the globin genes, reducing or preventing synthesis of one or more globin chains. The α-thalassemias, characterized by reduced α-globin synthesis, are generally caused by deletions that partially or completely remove one (-α) or both (--) α genes in the haploid genome or, more rarely, the α-globin major regulatory element (HS-40). They are classified as either α^+^, when there is partial synthesis of α chains, or α^0^, when there is no production of these chains (Bunn and Forget, 1986; Harteveld and Higgs, 2010; Galanello and Cao, 2011).

Individuals that are heterozygous for α^+^-thalassemia (-α/αα) have minimal or no hematological changes, while individuals homozygous for α^+^-thalassemia (-α/-α) and heterozygous for α^0^-thalassemia (--/αα) show moderate microcytosis and hypochromia. The presence of only one functional α gene (--/-α) results in chronic, moderate or severe hemolytic anemia, jaundice and hepatosplenomegaly, a condition known as Hb H disease. Homozygosity for α^0^-thalassemia (--/--) leads to Hb Bart's hydrops fetalis syndrome with severe tissue hypoxia. Without medical intervention it is incompatible with life and leads to intrauterine or early neonatal death (Bunn and Forget, 1986; Harteveld and Higgs, 2010; Galanello and Cao, 2011).

α-Thalassemia is estimated to affect around 5% of the population worldwide, with the -α^3.7^ deletion being the most common alteration (Piel and Weatherall, 2014). In Brazil, its prevalence is high (Sonati *et al.*, 1991; Couto *et al.*, 2003; Adorno *et al.*, 2005; Wagner *et al.*, 2010; Cardoso *et al.*, 2012; De Medeiros Alcoforado *et al.*, 2012). However, Hb H disease, which is found primarily in Southeast Asia, the Middle East and the Mediterranean, has only rarely been reported in Brazil, where most cases are the result of an interaction of the -α^3.7^ deletion with the --^MED^, -(α)^20.5^, or --^SEA^ deletions (Sonati *et al.*, 1992; Wenning *et al.*, 2000, 2002, 2009; Kimura *et al.*, 2009). Combinations of the -α^3.7^ deletion with new or rare α^0^ deletions started to be detected in the Brazilian population more recently, suggesting that the prevalence of Hb H disease may be underestimated (Suemasu *et al.*, 2011).

The deletions that most commonly cause α-thalassemia in populations [-α^3.7^, -α^4.2^, -(α)^20.5^, --^MED^, --^SEA^, --^FIL^, --^THAI^] are easily detected by multiplex gap PCR (Chong *et al.*, 2000), a technique that can only be used to screen known deletions. Multiplex ligation-dependent probe amplification (MLPA) is a sensitive technique that allows relative quantification of target regions in the genome and can be used to detect gene deletions and duplications and estimate their lengths (Schouten *et al.*, 2002; Harteveld *et al.*, 2005; Stuppia *et al.*, 2012). MLPA was used here to investigate the molecular basis of α-thalassemia in five unrelated patients, three of whom had Hb H disease.

This study was approved by the Research Ethics Committee at the School of Medical Sciences, Unicamp (CEP/FCM/Unicamp) under reference number 918/2007 dated February 18, 2007. All patients or responsibles gave their written consent.

Five unrelated patients (P) with suspected thalassemia were referred to our laboratory for investigation. Three of them had Hb H disease, while the other two presented with microcytosis, hypochromia and normal Hb A_2_ levels without iron deficiency. Familial analysis could only be carried out for four patients (Tables 1 and 2).

**Table 1:** Hematological and molecular data of Patients P1 - P3 and their families, and P4.

Cases	P1	MP1	FP1	BP1	P2	MP2	FP2	BP2	P3	DP3	P4
Age/Gender	17/M	42/F	42/M	14/M	35/F	-/F	-/M	-/M	58/F	24/F	22/F
RBC (10^6^/mm3)	5.43	4.79	6.61	4.94	5.50	5.72	3.94	5.64	5.50	5.60	5.00
RV: M: 4.5-6.1/F: 4.2-5.4											
Hb (g/dL)	8.8	12.4	13.5	12.3	11.2	11.8	13	11.7	12.5	11.8	8.3
RV: M: 14-18/F: 12-16											
MCV (fL)	56	80.2	66.9	77.7	65.3	68.2	100.8	66.7	70.4	65.4	59.4
RV: M: 81-99/F: 80-96											
MHC (pg)	16.2	25.9	20.4	24.9	20.4	20.6	33	20.7	22.4	21.1	16.6
RV: 27-32											
Hb profile	A_2_,A,H	A_2_,A	A_2_,A	A_2_,A	A_2_,A	A_2_,A	A_2_,A	A_2_,A	A_2_,A	A_2_,A	A_2_,A,H
Hb A_2_ (%)	1.6	2.9	2.4	2.8	2.6	2.4	2.6	2.7	2.5	-	0.8
RV: 1.5- 3.5											
Hb F (%)	0.1	0.3	0.2	0.3	0.6	0.7	0.3	0.6	0.4	-	1.7
RV: < 2											
α-Genotype	--/-α^3,7^	-α^3,7^/αα	--/αα	-α^3,7^/αα	--/αα	--/αα	αα /αα	--/αα	--/αα	--/αα	--/-α^3,7^

P: patient; MP: mother of the patient; FP: father of the patient; BP: brother of the patient; DP: daughter of the patient; M: male; F: female; RBC: red blood cells; RV: reference values; Hb: total hemoglobin; MCV: mean corpuscular volume; MCH: mean corpuscular hemoglobin.

**Table 2:** Hematological and molecular data of Patient P5 and her family.

Cases	P5	MP5	FP5	BP5	BP5	SP5	SP5	BP5	BP5
Age/Gender	2m/F	40/F	46/M	10/M	13/M	18/F	19/F	20/M	22/M
RBC (10^6^/mm3)	4.64	5.41	5.51	5.26	5.97	5.34	5.47	5.33	5.49
RV: M: 4.5-6.1/F: 4.2-5.4									
Hb (g/dL)	8.1	14.3	12.8	8.9	9.9	11.3	11.4	16.2	14.5
RV: M: 14-18/F: 12-16									
MCV (fL)	62.3	81.9	72.4	61.8	59.6	68.9	66.7	89.1	83.6
RV: M: 81-99/F: 80-96									
MHC(pg)	17.5	26.4	23.2	16.9	16.6	21.2	20.8	30.4	26.4
RV: 27-32									
Hb profile	A_2_,A,H, Bart's	A_2_,A	A_2_,A	A_2_,A,H	A_2_,A,H	A_2_,A	A_2_,A	A_2_,A	A_2_,A
Hb A_2_ (%)	1.0	2.5	1.7	1.6	1.8	2.7	2.7	2.7	0.1
RV: 1.5- 3.5									
Hb F (%)	22.6	0.2	0.3	0.1	0.1	0.2	0.1	0	2.2
RV: < 2									
α-Genotype	--/-α^3,7^	-α^3,7^/αα	--/αα	--/-α^3,7^	--/-α^3,7^	--/αα	--/αα	αα /αα	-α^3,7^/αα

P: patient; MP: mother of the patient; FP: father of the patient; BP: brother of the patient; SP: sister of the patient; m: months; M: male; F: female; RBC: red blood cells; RV: reference values; Hb: total hemoglobin; MCV: mean corpuscular volume; MCH: mean corpuscular hemoglobin.

A Sysmex hematology analyzer (Sysmex XE2100, Sysmex, Kobe, Japan) was used for cell counts and hematological indices; cation-exchange high-performance liquid chromatography (HPLC) (Variant, Bio-Rad Laboratories, Inc., Hercules, CA, USA) and electrophoresis on cellulose acetate in neutral and alkaline pHs were used in the hemoglobin analysis. Hb H inclusion bodies were observed in the three patients with Hb H disease after whole blood was incubated with brilliant cresyl blue (Dacie and Lewis,1995).

Genomic DNA was extracted from peripheral blood leukocytes using a commercial kit (QIAamp DNA Blood Mini Kit, *Qiagen*® GmbH, Hilden, Germany), and multiplex-gap-PCR was used to screen for the seven most common α-thalassemia deletions [-α^3.7^, -α^4.2^, -(α)^20.5^, --^MED^, --^SEA^, --^FIL^, --^THAI^] (Chong *et al.*, 2000). A search for the most prevalent non-deletional mutations (α^HphI^α, α^NcoI^α, αα^NcoI^, α^TSaudi^α) was carried out after selective amplification of the α genes, followed by analysis with the respective restriction enzymes, or, in the case of T^Saudi^, a specific nested PCR (Kattamis *et al.*, 1996). The -α^3.7^ deletion was only detected in the three patients with Hb H disease. Hematological and molecular data for the five patients are shown in Table 1.

To identify the α^0^ deletions, MLPA was performed with the SALSA MLPA P140 C1 HBA kit (MRC-Holland, Amsterdam, The Netherlands), which allowed to examine the region extending from the telomeric region of chromosome 16p to the DECR2 gene (approximately 360 kb of DNA). Comparative analysis of the fragments was performed using the Coffalyser. Net software to evaluate possible changes in the number of copies of the α locus in the samples.

In the three cases with Hb H disease (P1, P4 and P5), -α^3.7^ was present in combination with the α^0^ deletion, while the two other cases were heterozygous for α^0^-thalassemia. P1 and P3 had the same pattern of deletions in MLPA, including probes 318 to 283 and the region between them, with a deletion extending from ψζ (the zeta pseudogene) to the downstream region of the α_1_ gene. P2 had a deletion restricted to the HS-40 region that involved probes 236 to 364 and left the genes in the α cluster structurally intact, while P4 had a deletion between probes 292 and 400 that also extended from the ψζ gene to the downstream region of the α_1_ gene. In P5, the deletion detected affected a larger fragment, extending from the telomere to part of the RGS11 gene and involving probes 463 to 472. The five deletions are schematically shown in Figure 1.

**Figure 1:**
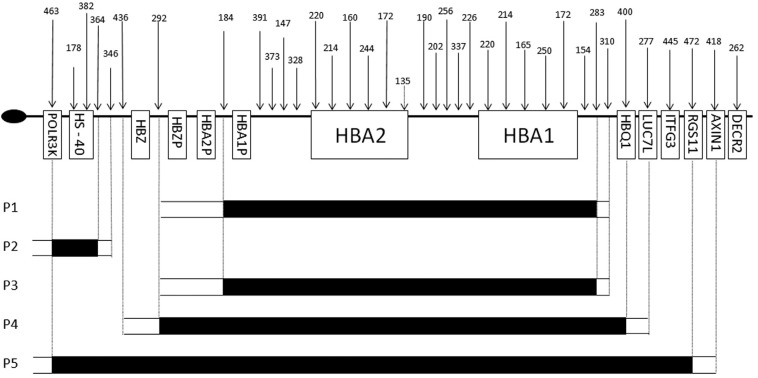
Schematic representation of chromosome 16p13.3. The oval represents the telomeric region, the arrows the locations of the probes and the boxes the genes. The black bars correspond to the deleted fragments, the dotted lines denote the first and last deleted probes delimiting the segments containing the breakpoints (adapted from MRC-Holland, provider of the MLPA kit).

Familial studies revealed the α^0^ allele in P1's father and the -α^3.7^ deletion in his mother and brother. P2 inherited the deletion in the HS-40 region from her mother, but no deletions were detected in her father. P3 has a daughter with the same molecular defect. P4's family was not available for familial analysis. P5's mother had the -α^3.7^ deletion, while the α^0^ allele was inherited from his father; of the patient's six siblings, one had the -α^3.7^ deletion, two sisters had the α^0^ deletion and two brothers also had Hb H disease. Only one member of this family of nine (one of the patient's brothers) did not have any deletions. Figure 2 shows the pedigrees of the four families studied.

**Figure 2:**
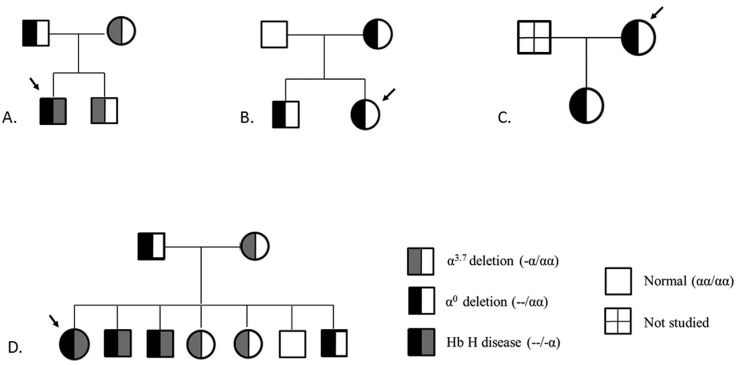
Pedigrees of the families studied. (A) P1 has Hb H disease (-α^3.7^ and α^0^ alleles), while his mother and brother are heterozygous for the -α^3.7^ deletion and his father for the α^0^ deletion. (B) P2 and her mother and brother are heterozygous for the α^0^ deletion, while her father has normal α-genotype (no deletion). (C) P3 and her daughter are heterozygous for the α^0^ deletion. (D) P5 and two of her brothers have Hb H disease (-α^3.7^ and α^0^ alleles); her mother and two sisters are heterozygous for the -α^3.7^ deletion, while her father and one of her brothers are heterozygous for the α^0^ deletion. Only one of the siblings does not have any deletions.

Multiplex-gap-PCR is the most widely used method to screen for α-thalassemia and can identify the deletions that most frequently affect populations worldwide. However, there are cases of patients with reduced MCV and MCH values, normal or reduced Hb A_2_ and Hb F and normal iron status in whom a diagnosis could not be reached. In these patients it is important to investigate rare or new deletions that affect the α genes and/or their regulatory elements. The combination of two α^0^ deletions results in Hb Bart's hydrops fetalis syndrome, while the association of these with more common deletions, such as the -α^3.7^ deletion, causes Hb H disease, a moderate or severe type of hemolytic anemia that may require blood transfusions and/or splenectomy. Even for heterozygotes, the correct diagnosis is extremely important, as microcytosis and hypochromia are frequently interpreted as indications of iron deficiency (Borges *et al.*, 2001) and incorrectly treated.

Patients P1 and P3 appear to have a 15 kb deletion (positions 162735-177934 according to the USCS Genome Browser, March 2006), which is similar to that described as --^GB^ by Harteveld *et al.* (2005) in a Dutch individual of mixed ethnic backgrounds, and found again, by Phylipsen *et al.* (2010), in three unrelated individuals of Arabic, Indian and unknown origin. Patient P2 has a deletion of approximately 97 kb that removes the α-MRE (positions 46407-143677 according to the USCS Genome Browser, March 2006); this is similar to the (αα)^MM^ deletion reported by Romão *et al.* (1991) in a child from the Azores, and by Wenning *et al.* (2002) in a Brazilian family. In P4, the deletion of about 22 kb (positions 159487-181215 of the USCS Genome Browser, March 2006) appears to be the same as that described by Phylipsen *et al.* (2010) in two unrelated individuals of Indian and unknown origin. P5 has a deletion of approximately 225 kb (positions 46407-271806 according to the USCS Genome Browser, March 2006) that affects the entire α cluster and the α-MRE, and is of similar length to that described by Suemasu *et al.* (2011) in a Brazilian patient. It is possible that the deletions found in the patients studied here are the same as those previously described, and that have they been introduced to Brazil by immigration. However, as their breakpoints have not yet been accurately identified, we cannot rule out the possibility that one or even all of these are new deletions.

With the development of new techniques and technologies, an increasing number of novel and rare deletions compromising both alpha genes have been detected, suggesting that their frequencies (and heterogeneity) may be underestimated in populations (Gilad *et al.*, 2014; Brieghel *et al.*, 2015; de-la-Cruz-Salcedo *et al.*, 2016; Hu *et al.*, 2016; Mota *et al.*, 2017; Wang *et al.*, 2017; Wu *et al.*, 2017). Our findings highlight the importance of using MLPA in the characterization of rare deletions, allowing the molecular basis of α-thalassemia to be elucidated when conventional methods fail. In addition to allowing correct diagnosis and treatment of carriers, characterization of these changes is important in genetic counseling, as it allows couples to understand the risk of having an affected child and to make an informed reproductive decision. Furthermore, knowledge of the size of these deletions and the genes and regulatory elements affected by them can greatly help to elucidate the genetic recombination mechanisms in the affected regions and the functions of the α-globin genes, about which little is yet known to date.
